# Supplementary material for: Are carbon emissions trading and green financial instruments synergistic? -Comprehensive quantitative research based on content analysis
Source: PLoS One. 2024 Mar 7;19(3):e0298601. doi: 10.1371/journal.pone.0298601 (PMC10919601; doi:10.1371/journal.pone.0298601)
Supplement: S1 Data — (ZIP) [file pone.0298601.s001.zip › Data/地级市Z_F工作报告.zip!/─┌├╔╣┼/╬┌└╝▓∞▓╝╩╨/└┤╫╘╣1⁄2╓┌║┼í╛┬φ┐╦╩2╛▌═°í┐_2019.docx]

各位代表：

　　现在，我代表市人民政府向大会报告工作，请予审议，并请市政协委员和列席会议的同志们提出意见。

　　一、2018年工作回顾

　　2018年是贯彻党的十九大精神开局之年，也是本届政府履职第一年。一年来，在自治区党委、政府和市委的坚强领导下，全市上下以习近平新时代中国特色社会主义思想为指导，全面贯彻党中央、国务院决策部署，牢牢把握稳中求进工作总基调，按照高质量发展要求，扎实推进稳增长、促改革、调结构、惠民生、防风险各项工作，经济社会持续健康发展，向全面建成小康社会的目标迈出了坚实步伐。全年地区生产总值增长5.5%，一般公共预算收入增长7.2%，社会消费品零售总额增长7.3%，城乡居民人均可支配收入分别增长7.6%和9.3%。主要指标增速位居全区中等水平。

　　新旧动能转换取得新突破。规模以上工业增加值增长6%，工业固定资产投资增速全区第一，占全部固定资产的45%，战略性新兴产业占工业总产值比重达到10%。大数据产业国内领先地位基本确立。苹果、华为二期、阿里巴巴等数据中心项目落地开工，总投资243亿元，服务器规模100万台，被评为“中国最适合投资数据中心的城市”。鸿联九五、博岳等服务外包企业运营坐席1500席，软通动力乐业空间在孵企业40家，百度创新中心等数据应用项目加快推进。“南贵北乌，草原云谷”成为乌兰察布的新名片，受到国务院通报表扬。大物流产业格局基本形成。天津港、曹妃甸港内陆港运行平稳，开行直达天津港的双向铁路班列，庙梁物流园区运销煤炭1330万吨。北方陆港取得俄蒙国际公路运输资格。中欧班列开行80列、开通5条国际线路，七苏木物流基地一期建成投运，配套建设的莫斯科和乌兰巴托物流园区具备运营条件。成功举办全国物流园区工作年会，被列为“国家物流枢纽布局载体城市”。大旅游产业初具规模。岱海国际滑雪场被评为国家4A级景区，集宁战役红色纪念园入选中国旅游产业影响力景区。成功举办中俄蒙美食文化节、草原音乐节、旅游那达慕等一批文旅活动。全年旅游接待人数和收入分别增长10%和15%，被评为“中国最美养生休闲旅游城市”。新能源新材料产业加快发展。风电、光伏装机容量新增52万千瓦，达到652万千瓦，600万千瓦风电基地示范项目获批。瑞盛一次电池导电剂达到国际领先水平，高导热石墨材料填补了国内空白，锂离子电池负极材料实现规模化、集群化发展，石墨烯实现小批量生产。海立、东阳光高压化成箔项目建成投产。吉铁百万吨合金、雄伟光大70万吨微碳合金项目投产运行，成为全国最大的绿色合金生产基地。氟化工循环经济产业集群不断壮大。PVA产能居全区第一。农牧业产业结构不断优化。粮食总产量22.4亿斤。冷凉蔬菜产量39亿斤。新增优质专用薯30万亩、达到180万亩，乌兰察布马铃薯入选首届“中国农民丰收节”农产品百强榜，列入“中国特色农产品优势区”。燕麦种植面积增加到100万亩，被命名为“中国燕麦之都”。大力发展节水农业，改造喷灌圈16万亩。建成标准化示范牧场39个，肉类产量达到28万吨。农畜产品加工转化率由59%提高到62%。“三品一标”认证产品达到243个，国家地理标志认证产品和证明商标分别达到9种、14件。

　　三大攻坚战取得新成果。防范化解重大风险初战告捷。采取财政预算安排、压缩一般性支出、盘活存量资产等措施，分类处置政府债务，完成年度化债任务的101.9%，“十个全覆盖”十项工程拖欠工程款全部清偿，欠薪问题得到有效解决。停建缓建瘦身了一批政府投资项目，全年未发生新增隐性债务。市委、市政府带头树立过“紧日子”思想，压缩市本级“三公”经费367万元。严厉打击非法集资、金融诈骗等违法犯罪活动，累计清收处置不良贷款18.28亿元。脱贫攻坚取得决定性进展。

各级财政累计投入扶贫专项资金42.5亿元，增长64.7%。5.9万贫困人口稳定脱贫，贫困发生率下降到1.79%，丰镇市脱贫“摘帽”，凉城县、察右后旗达到退出标准。建成脱贫车间70个，16.6万千瓦集中式光伏扶贫电站并网发电，1321个嘎查村有了集体经济收入，产业扶贫惠及11.2万贫困人口。建设易地搬迁安置房1.63万套，危房改造5.82万户。深化京蒙扶贫协作，互派挂职干部56人，落实援助资金3.2亿元，引进大北农等产业项目32个，完成投资20亿元。中国银保监会等7个中直单位、自治区金融办等64个区直单位定点帮扶效果显著。健康扶贫政策进一步完善，贫困户大病和慢性病报销比例达到90%。1.5万贫困家庭学生得到资助，9.43万贫困人口纳入低保。污染防治成效明显。深入推进环保督察突出问题专项整改，生态环境质量持续改善。《岱海黄旗海保护条例》颁布实施，《岱海水生态保护规划》修编完成。“两节两补两恢复”综合治理措施扎实推进，岱海电厂停止取排湖水，湖面缩减趋势得到遏制。地表水国控断面达到国家考核要求，大黑河流域实现废水零排放，建成区黑臭水体基本消除。累计恢复治理矿山56.28平方公里，白乃庙煤矿灭火工程顺利完成。工业固废综合利用水平逐步提高。空气质量综合评价全部达标，中心城区优良天数达到310天。生态保护红线划定基本完成。天蓝、地绿、水清的良好生态环境，成为乌兰察布的亮丽底色。

　　改革开放取得新成效。“三去一降一补”深入推进。国有担保体系实现旗县市区全覆盖，支持重点项目和企业贷款67.5亿元。完成钢铁产能置换60万吨。商品房库存下降28.4%。盘活闲置低效工业用地5829亩。减轻企业税费负担16.7亿元，降低用电成本12亿元。“放管服”改革持续深化。全面推行“多证合一”“证照分离”改革，减少各类证明事项157项。推行“一个窗口对外、一站式审批”和“最多跑一次”，企业开办时间压缩至5个工作日内，行政审批按时办结率100%。试运行“互联网+政务服务”平台，开发“乌立即”指尖政务小程序，“五个一”工程建设审批体系逐步完善，“一网通办”取得新进展。深入整治“红顶中介”，加强“双随机一公开”监管。市场主体每个工作日新增93户，全年累计突破12万户。营商环境综合评价全区排名第三，被评为“2018年中国最佳表现城市”。农村牧区改革稳步实施。土地确权、“两区”划定、农垦改革基本结束，1352个嘎查村清产核资全部完成。开放合作成果显著。积极承接京津冀产业转移，深化乌大张区域合作，深入对接长三角、珠三角等地区，全年实施招商引资项目190项，完成投资178亿元。出台促进外经贸快速发展扶持政策，与乌兰乌德、乌兰巴托签署合作协议，成功举办中蒙俄特色商品博览会。全市外贸进出口总额11.8亿元，被评为“中国外贸百强城市”。

　　城乡建设开创新局面。创城工作深入开展，国家食品安全示范城市创建进入全面验收阶段，察右前旗被评为自治区园林县城。编制完善特色小镇、园区建设等专项规划，城市设计和控规覆盖率达到80%以上，人均道路面积、污水处理率等指标进入自治区前列。改造棚户区2.9万套，恒大、富力等一批高品质商住项目落地建设。中心城区南沙河综合治理成效显著，取消无电子计费系统停车场收费,公交刷卡实现与呼市“双城一卡通”。美丽乡村建设不断升级，制定出台乡村振兴战略实施意见和规划，全面开展农村牧区人居环境整治和乡风文明提升行动，建设垃圾池8000多个、公厕2000多座，创建8个全国文明村镇。隆胜、七家营等水库基本建成，安全饮水工程惠及3.7万农牧民。建设“四好农村路”255公里，99%的嘎查村开通客运班车。生态和基础设施建设持续加强，集宁至科布尔一级公路建成通车，集大高铁项目顺利推进，呼张客专集宁至张家口段完成铺轨。实施电网工程32项，新增变电容量25.6万千瓦。全部乡镇和部分嘎查村实现光纤入户，第二条直通北京的专用光缆即将投用。实施京津风沙源治理54.8平方公里，完成人工造林43万亩，其中经济林10.9万亩。

　　人民生活得到新改善。财政用于民生社会事业的支出达到83.5%。城镇新增就业1.7万人，农牧民转移就业37.8万人。成功举办第三届中国创业创新博览会和自治区“双创”活动周，新增自治区级众创空间和星创天地5家、国家高新技术企业16家，专利申请和授权量分别增长63.5%和102.8%。深化教育“三项改革”，完成校舍建设38万平方米，普惠性幼儿园覆盖率达85%，高中阶段办学水平明显提升，教育事业得到长足发展。公共文化体育场馆向社会开放，嘎查村综合文化服务中心实现全覆盖，成功承办“十四运”、第五届残运会、首届智力运动会等自治区级赛事。在全区率先出台加快推进乌兰牧骑事业发展实施办法。文献纪录片《集宁战役》开拍，反映草原人民收养上海三千孤儿的电视剧《国家孩子》进入后期制作。二级以上医院完成150个单病种付费改革，旗县医共体增加到10个，重点人群家庭医生签约率达70%。加强公共卫生防控，有效应对非洲猪瘟等疫情。养老保险实现制度全覆盖，城乡医疗保障体系不断完善。43万城乡低保对象、10余万孤老残幼等特困群体纳入社会保障。社会治安综合治理体系更加完善，扫黑除恶专项斗争深入推进，一批信访积案得到解决，连续三年实现生产安全事故起数和死亡人数双下降，平安乌兰察布建设取得实效。

　　全面贯彻党的民族宗教工作基本方针，民族团结进步教育和创建活动深入开展。妇女、儿童、残疾人事业稳步发展，审计、统计、地震、气象、人防等工作不断加强，双拥共建和国防建设扎实推进。

　　政府自身建设取得新进展。坚持把党的领导贯穿于政府工作始终，持续推进“两学一做”学习教育常态化，深入开展“贯彻落实习近平新时代中国特色社会主义思想，担当作为谋发展，尽职实干促跨越”大学习大讨论活动，大力弘扬求真务实、真抓实干、敢于担当的优良作风。加强政府系统党风廉政建设，严格执行“三重一大”制度，强化审计监督，推进政府预算、部门预算、三公”经费向社会公开，“营造干部清正、政府清廉的良好政治生态。强化专项督查，狠抓重大政策落地，深入推进巡视反馈意见整改落实。严格执行市人大及其常委会决议决定，自觉接受人大、政协和社会监督，人大代表议案、建议和政协委员提案办结率达到100%。

　　各位代表！

　　过去一年，面对复杂多变的宏观形势和艰巨繁重的发展任务，我们知难而进求突破，锲而不舍抓落实，战胜了各种困难和挑战，取得了来之不易的成绩。一年的实践启示我们：在新时代推动新跨越、开创新未来，最根本的是绝对忠诚，必须树牢“四个意识”，坚定“四个自信”，坚决做到“两个维护”，不忘初心，牢记嘱托，把忠诚刻进心坎里、写在岗位上、融入事业中；最关键的是抓好发展，必须紧紧抓住发展第一要务，坚持以经济建设为中心，持续加快新旧动能转换，扎实推动高质量发展；最核心的是人民至上，必须始终把以人民为中心的发展思想贯穿到政府全部工作中，为民办事、为民解忧，不断增进民生福祉，让人民群众最大限度分享改革发展成果；最可贵的是担当作为，必须勇于任事、敢于担事，面对矛盾不绕行，面对困难不退缩，面对压力不懈怠，同心同向把乌兰察布各项事业推向前进。

　　2018年是改革开放40周年。40年来，全市各族干部群众艰苦奋斗、开拓创新，经济社会发展和人民生活发生了翻天覆地的巨大变化。这是以习近平同志为核心的党中央关怀厚爱的结果，是自治区党委、政府和市委坚强领导的结果，是市人大、政协监督支持的结果，是全市各族干部群众团结奋斗的结果。在此，我代表市人民政府，向全市各族人民，向所有关心支持乌兰察布发展的各界人士，致以衷心的感谢！

　　成绩的背后，我们也面临诸多“两难”选择，既要去产能、去杠杆，又要守底线、稳就业，生态要保护、发展要提速，债务要化解、民生要保障，各种矛盾和问题交织叠加,挑战和风险明显增多。这既有宏观环境的影响，也有我们自身存在的问题，有些是长期积累形成的，有些是新出现的。主要表现在，经济下行压力较大，项目储备不足，固定资产投资下降明显；产业结构不优的矛盾依然突出，实体经济困难和金融风险并存；群众在上学、看病、供暖、物业服务等方面，还有不少堵点、难点；政策落实存在“中梗阻”“最后一公里”“一刀切”等问题，政府工作还有许多不到位、不及时、不如人意的地方，一些单位作风漂浮、工作不实，部分干部墨守成规、不敢担当，与新时代新任务新要求不相适应。对此，我们必须保持清醒头脑，增强发展定力，抓住主要矛盾，奋发有为抓好各项工作，决不辜负全市人民的期望。

　　二、2019年总体思路和重点任务

　　2019年是新中国成立70周年，是全面建成小康社会、实现第一个百年奋斗目标的关键之年。按照市委四届七次全会精神，今年政府工作的总体思路是：以习近平新时代中国特色社会主义思想为指导，全面贯彻党的十九大和十九届二中、三中全会精神，统筹推进“五位一体”总体布局，协调推进“四个全面”战略布局，坚持新发展理念，以供给侧结构性改革为主线，加快推动高质量发展，坚决打好三大攻坚战，做强大物流、大旅游、大数据等特色主导产业，着力打造优质高效的营商环境和安全稳定的社会环境，抓招商、抓项目、抓民生、抓作风转变，统筹推进稳增长、促改革、调结构、惠民生、防风险工作，打造自治区开发开放新高地，推动经济持续健康稳定发展，为全面建成小康社会收官打下决定性基础，以优异成绩庆祝中华人民共和国成立70周年。

　　主要预期目标是：地区生产总值增长6%左右，固定资产投资增长8%左右，社会消费品零售总额增长6.5%左右，城乡居民可支配收入分别增长7.5%和8%左右，城镇新增就业1.4万人以上，城镇登记失业率控制在4.5%以内。完成国家下达的年度节能减排任务目标。

　　做好今年工作，我们要认真贯彻习近平总书记对内蒙古提出的“四个着力”“三个扎实”重要要求，落实好李克强总理考察我市重要指示精神，按照中央经济工作会议、自治区党委十届八次全会暨全区经济工作会议、自治区十三届人大二次会议及市委四届七次全会精神，坚持“巩固、增强、提升、畅通”八字方针，深化供给侧结构性改革，坚定不移抓机遇、用机遇，立足优势创造性开展工作，不断开创新时代高质量发展新局面。

　　（一）坚决打好“三大攻坚战”，夯实高质量发展基础

　　全面防范化解重大风险。牢固树立底线思维，增强忧患意识，提升风险的发现力、研判力、处置力，统筹防范化解政治、意识形态、经济、社会等领域风险隐患。按照“坚定、可控、有序、适度”的要求，把政府债务纳入预算管理，积极争取新增专项债券，稳妥推进市场化债转股，加快国有资产整合重组，推动公共服务设施市场化运营，严格按照时限要求化解政府债务，坚决遏制隐性债务增量。坚持量力而行，规范政府举债行为，严格实行政府投资项目债务风险评估和合法性审查，推动融资平台公司市场化转型。加大不良贷款处置力度，开展网络借贷、企业债券违约等风险点整治。扎实开展矛盾纠纷大排查、隐患大整改、治安大整治，防范化解社会稳定、公共安全、网络安全等各类风险，坚决守住不发生区域性、系统性风险的底线。

　　决战决胜脱贫攻坚。聚焦“两不愁三保障”突出问题，贯彻“精准、可持续、激发贫困群众内生动力和自我发展能力”三个关键要求，实施好“清零达标”专项行动，确保7个国贫旗县“摘帽”、剩余贫困人口全部脱贫。强举措攻难点。突出抓好中央专项巡视反馈意见整改落实，确保脱贫成效经得起实践和历史检验。全面完成农村牧区危房改造，扎实推进易地搬迁和小村整合，实现搬迁后村村有产业、户户能脱贫。实施安全饮水工程574项，确保建档立卡贫困户饮水安全全部达标。统筹做好健康、教育、生态、社会保障兜底扶贫。强产业扶长远。优化规范脱贫攻坚项目库建设，精准实施脱贫车间、村级电站等扶贫措施，完善贫困户和龙头企业紧密型利益联结机制，实现贫困人口产业全覆盖，集体经济收入低于5万元以下现象清零。加大与电商平台合作力度，以“互联网+扶贫”模式推进消费扶贫。坚持智志双扶，多渠道推动贫困人口转移就业，提升自我发展意识和能力。强协作聚合力。深入落实京蒙扶贫协作三年行动框架协议，推进“万企帮万村”精准扶贫行动，在北京设立特色农畜产品店、举办展销活动。发挥中央和区直单位定点帮扶优势，落实好金融支持和倾斜特惠政策，创建金融扶贫示范区。加快推进深度贫困旗县拆旧复垦项目建设，积极争取扩大城乡建设用地增减挂钩交易面积。强监管求实效。发扬“精、准、严、实”的作风，集中力量解决责任落实不到位、工作措施不精准等问题。全面加强扶贫资金使用管理，鼓励引入第三方监督，探索建立协同管理机制。加强扶贫对象动态管理，严把贫困退出关。建立完善贫困边缘户帮扶救助政策，减少致贫、防止返贫。

　　全力推进污染防治。牢固树立绿色发展理念，统筹山水林田湖草生态修复治理，增加环境“含绿量”，提升发展“含金量”。坚决整治突出问题。按照坚守阵地、巩固成果的要求，狠抓中央环保督察整改工作，坚决不要“带灰的GDP”。持续推进岱海水生态综合治理，深入实施“两节两补两恢复”工程，全面完成岱海电厂空冷改造。加快推进“引黄济岱”前期工作，从根本上解决岱海水生态问题。严格落实河湖长制，加快黄旗海等流域治理，切实保证黑河、大黑河国控出水断面水质达标。加强各类园区污水处理设施建设，提标改造集宁区等6个污水处理厂。加快工业园区渣场建设，推动工业固废资源化综合利用。加大自然保护区内工矿企业治理力度，严厉打击违法违规征占草原、河道采砂等行为。实行浓度达标和总量许可双重管理，加大火电企业超低排放改造力度，严格控制高耗能企业大气污染物排放。抓好畜禽粪污资源化利用，全面治理农业面源污染。持续推进生态建设。全面落实新一轮草原生态奖补政策，严格执行禁牧、草畜平衡等制度，加大退化草原治理力度。深入推进国土绿化行动，实施京津风沙源治理等国家重点生态工程，统筹城镇、村屯、通道、园区等重点区域绿化。积极开展全民义务植树和万亩生态园建设，大力发展特色经济林，全年完成新造林40万亩。形成绿色发展长效机制。全面推行领导干部自然资源资产离任审计。建立生态保护红线监管平台，实现一条红线管控重要生态空间。坚持量水而行，以水定城、以水定产，实施最严格的水资源管理制度，实行地下水超采区水量定额控制。全面推行农业水价综合改革，加快发展节水农业、旱作农业，建设高效节水灌溉田30万亩。严把项目环保审批关，抓好企业节能减排降耗，推动低碳循环发展。在四子王、凉城等旗县推行耕地轮作试点，面积扩大到100万亩以上。建设10座自治区级绿色矿山，开展节约型机关、绿色家庭、绿色企业等创建活动，让广大群众共享绿色发展红利。

　　（二）调整优化产业结构，培育高质量发展新动能

　　打造大数据产业链。把大数据作为推动高质量发展的战略引擎，加快数据中心项目建设投运，大力发展服务器制造、金融保险行业数据应用等上下游产业，推进大数据向各领域广泛渗透、与各行业深度融合，建设国家大数据云计算产业基地。推动大数据与实体经济深度融合。实施“万户企业登云”三年行动计划，建设一批产业大数据云平台，运用大数据手段改造升级传统产业。大力发展通用航空产业，引进国内领先的无人机、有人机制造企业和各类航空学院、培训机构，加快推进航空产业园和飞行测试基地等项目落地，积极开发农业植保、消防等无人机应用市场，打造具有整体竞争优势的智能制造产业集群。推动大数据与社会治理深度融合。进一步深化“互联网+政务服务”，推动市县两级政务数据资源整合共享，加快构建全市数据资源库。统筹推进政府网站、实体政务大厅的线上线下联通，实现数据同源、服务同根。优先推进交通、医疗、社保、教育等领域政府数据向社会开放，鼓励和支持社会力量参与政府数据资源开发利用，加快建设“数字政府”。推动大数据与服务民生深度融合。加强政务新媒体建设，普及腾讯“为村”微信公众号，完善“乌立即”小程序，推动更多民生事项实现“数据替跑”掌上即办”，让“查”的功能越“来越全、办”的项目越来越多。拓宽大数据民生服“务应用范围，推广扫码坐公交、取快递等智能化服务，把大数据融入百姓生活方方面面。

　　培育大物流产业集群。充分发挥区位交通优势，加快陆港型物流枢纽载体城市建设，大力发展物流贸易加工，开启乌兰察布“枢纽经济”新时代。依托中欧班列，做大“白货”物流加工基地。加快北方陆港国际物流中心、七苏木国际物流园等物流园区建设，开展国际贸易分拨、集散、加工、结算等业务。提升境外物流园区功能，通过“海外仓”集货揽货，提高回程货运量。积极推进与中轻集团、天津物产等大型物流贸易企业合作，支持北方家居产业园等园区建设，大力发展木材、纸浆等物流加工业，提高物流附加值。提升互联互通水平，做强“黑货”物流加工基地。依托庙梁物流园区，汇聚大同、鄂尔多斯、锡林郭勒以及蒙古国等地煤炭资源，壮大煤炭洗选、混配、集散和深加工产业。加快推进园区—满都拉口岸铁路项目，支持易大宗等煤炭物流项目建设，打造华北最大的煤炭集聚加工基地、环渤海动力煤配送储备基地。壮大专业市场，建设华北物流集散中心。以红星美凯龙、集宁国际皮革城等专业市场为龙头，加快培育特色化、集群化的汽贸汽配、建材、药材等专业贸易市场，增强集散功能，扩大辐射半径，打造华北地区重要的中转、仓储、物流集散中心。

　　推动大旅游多元化发展。围绕“生态草原·避暑之都·冰雪乐园”旅游品牌，做好迎接高铁时代的准备，大力发展全域旅游、四季旅游，促进旅游与文化、体育、养老等产业融合发展，带动消费升级、拉动投资增长。全面推进景区提档升级。格根塔拉、林胡古塞等旅游区列入自治区创建5A级景区名录，辉腾锡勒旅游区通过国家文旅部旅游资源评价，火山草原旅游区创建国家研学旅游示范基地。推进红召九龙湾、苏木山等景区旅游公路建设，加快旅游厕所、标识标牌、游客集散咨询中心等配套设施建设，完善房车营地、共享汽车等落地自驾保障体系。增强旅游参与度和体验感。深入挖掘历史文化资源，重点打造察哈尔文化、敕勒川文化等文化品牌，发展文化体验游。引进飞行体验、空中游览等项目，加快培育低空旅游消费市场。发展休闲式、体验式、养生式乡村旅游。

深入挖掘集宁战役、绥蒙革命、红格尔图战役等资源，大力发展红色旅游。依托岱海、大河湾等滑雪场，办好各类冰雪赛事活动，打造国家级冰雪运动基地、冰雪旅游集聚区。加强与俄蒙旅游合作，举办“三乌”城市汽车越野拉力赛，积极发展边境体验游。扩大高质量、个性化旅游精品供给，提升“吃住行游购娱”服务水平，让游客“玩得好、留得住、还想来”。提升旅游知名度和美誉度。加大城市旅游形象宣传力度，办好草原旅游那达慕、蒙古族服装服饰艺术节、草原音乐节等品牌活动，打造国内知名的特色旅游目的地、全域旅游示范区。全年接待游客突破1930万人次，旅游收入达到200亿元。

　　建设新能源新材料产业基地。加快推动集宁区和察右前旗热电联产项目建设，实施好商都200万千瓦风电项目，建设好乌兰察布600万千瓦风电基地示范项目，打造保障首都、服务华北、面向全国的清洁能源输出基地。扩大负极材料和石墨电极产业规模，加快产品创新和研发应用，打造北方地区重要的石墨新材料基地。加快推进吉铁300万吨、雄伟光大百万吨合金项目投产达效，培育绿色合金新材料产业集群。

　　推动传统产业延伸升级。围绕解决“四多四少”问题，调结构、优布局、强产业、延链条，提升传统优势产业核心竞争力。加快三爱富万豪、永和氟化工等项目建设，培育自治区重要的精细氟化工产业集群。扩大氯碱化工中高端产能，发展特种纤维等高附加值产品。鼓励发展新型墙材等绿色环保建材，推广绿色建筑和装配式建筑。

　　（三）实施乡村振兴战略，推进农业农村高质量发展

　　调整优化农牧业结构。坚持质量兴农、绿色兴农、品牌强农，深化农牧业供给侧结构性改革，推动农牧业由增产导向转向提质导向，切实提升全要素生产率。以打造种薯强市为目标，加强对马铃薯产业发展的战略谋划，在产业布局、结构调整、品种改良、生产加工、市场销售、龙头带动、品牌打造等方面进行系统研究，做好马铃薯产业这篇大文章。实施好国家马铃薯主粮化试点工作，落实好各项补贴政策。年内建设种薯田80万亩，优质专用薯200万亩，力争微型薯生产能力达到40亿粒。支持凯达、中加、土豆集等龙头企业发展，加快建设马铃薯加工产业园，打造华北地区最大的马铃薯交易平台，做强“乌兰察布马铃薯”品牌，推动马铃薯全产业链发展。以设施蔬菜为重点，巩固建设察右中旗红胡萝卜、商都西芹等九大特色蔬菜种植基地，培育壮大“两带一园”设施果蔬基地，在察右前旗、兴和集中打造万亩设施蔬菜园区，冷凉蔬菜面积达到75万亩。以特色有机为抓手，培育壮大龙头企业，大力发展藜麦、中药材等特色种植，燕麦杂粮杂豆达到200万亩。坚持“稳羊增牛扩猪禽”，加快建设肉羊标准化养殖场，打造绿色高效的草原肉羊示范区，扩大肉牛和生猪养殖规模，发展马驴驼等特色养殖，牲畜存栏稳定在800万头只。提升农畜产品附加值，以“中央厨房”和设施农业为重点，引进培育一批产业化龙头企业，提升农畜产品就地加工转化水平。完善冷链物流体系，加大储窖、恒温库和批发市场建设力度。加强与大型电商企业合作，用好“公益乌兰察布”等电商平台，加快推进电子商务进农村，让优质农畜产品卖出好价钱，培育打造一批畅销全国的“金字招牌”。

　　加快建设美丽乡村。突出抓好农村牧区人居环境整治，以垃圾污水处理、厕所革命和村容村貌提升为重点，实施“三清一改”村庄清洁行动，开展美丽宜居村庄和最美庭院创建活动。大力培育田园综合体等现代农牧业新模式，建设一批特色小镇、乡村民宿、牧人之家，每个旗县市区至少打造一个乡村旅游特色示范区。健全村庄基础设施建管长效机制。创新乡村治理体系，形成自治为基、法治为本、德治为先的治理格局。开展平安乡村创建行动，严厉打击“村霸”、宗族恶势力。

　　深化农村牧区改革。优化科技特派员队伍结构，推进主要农作物生产全程机械化，加强面向小农户的社会化服务，支持农牧民合作社和家庭农牧场发展。深化农村土地制度改革，全面完成土地确权合同签订、证书发放等工作，进一步完善承包地“三权”分置制度。完成农村牧区集体产权制度改革。清理整治“大棚房”，坚决遏制农地非农化。加快农村牧区产权交易中心建设，完善农牧业支持保护政策，优化乡村振兴制度环境。

　　（四）全面推进改革开放，激活高质量发展新动力

　　以优化营商环境、壮大民营经济为导向，进一步深化改革、扩大开放，加大招商引资和项目建设力度，激发市场活力，增强发展后劲。

　　坚定不移支持民营经济发展。开展“优化民营经济发展环境年”活动，紧盯市场主体期待和需求，加快打造市场化、法治化、国际化的营商环境，增强企业发展信心和竞争力。落实好“更大规模减税、更明显降费”政策。加大乱收费查处整治力度，切实降低民营企业用能、用地、物流等要素成本。支持企业创新发展，鼓励品牌创建和质量提升。有效缓解融资难融资贵问题。完善市场化运作的融资担保体系，拓展直接融资渠道。健全金融机构监管评价机制，提高民营企业贷款占比和覆盖面，纾解企业流动性困境。推进县域金融工程试点，实施农村信用社改革。借力京蒙扶贫协作，积极引进金融机构、投资机构，精准对接民营企业和小微企业融资需求。营造公平竞争环境。切实转变政府职能，持续深化“放管服”改革，大幅减少政府对资源的直接配置。扎实做好第四次全国经济普查。建立电子证照库，推进非税收入收缴电子化，持续开展减证便民行动。完善政务服务大厅功能，70%的服务事项只进一扇门、实现一次认证，工程建设项目审批时间缩减一半以上。抓好诚信政府建设，兑现好优惠政策，说到的就一定要做到。保障企业家合法权益。严禁行政执法机关以查处经济纠纷为由，干预企业正常经营活动。坚决纠正“一刀切”式执法，规范自由裁量权，防止任意检查、执法扰民。依法保护民营企业家人身和财产安全。积极解决企业用工、住房、子女入学和落户等具体困难，做到“有求必应，无事不扰”。

　　坚定不移扩大对内对外开放。围绕“打造全区开发开放新高地”的目标要求，以高水平开放推动高质量发展。深度参与“一带一路”中蒙俄经济走廊建设。发挥口岸腹地城市比较优势，依托二连浩特国家开发开放试验区，建设辐射自治区中部地区的集宁物流中心。大力发展泛口岸经济，加快设立B型保税物流中心、航空口岸，建设国际快件库、中蒙俄产业园等项目，推动通道经济向落地经济转型。加强与俄蒙基础设施互联互通合作，加大“三乌”通道建设力度，加快推进过境列车换轮，争取开通蒙古、俄罗斯等国航线。落实与蒙古国东戈壁省、俄罗斯布里亚特商会合作协议，积极引进俄蒙欧企业，提升对外贸易发展水平，力争进出口总额实现翻番。办好第四届中俄蒙三国旅游部长会议、第三届中蒙博览会、中俄蒙美食文化节等会展活动。深度融入京津冀协同发展。以京呼高铁开通为契机，充分调动政府、市场、社会力量，加快推动城市公共设施、酒店服务、人才引进、消费环境大整合大提升。高标准建设京蒙合作产业园，做好与北京项目、产业、政策的衔接争取，大力发展“飞地经济”。加快集大高铁征地、拆迁、资金等前期准备工作，推动项目早日开工建设。规划建设好高铁车站及周边沿线区域，把京呼、集大“高铁经济走廊”培育成新的经济增长极。积极打造乌大张合作发展示范区。深度推进与呼包鄂协同发展，做好规划对接和政策衔接，推动基础设施互联互通，营造人才、资本、技术等要素汇聚高地，共同打造自治区现代化经济体系承载区和高质量发展新引擎。

　　加大招商引资和项目建设力度。发挥投资关键作用，力争年内开工100个、投产100个、储备100个产业项目。实行精准招商。以“招商引资突破年”为主题，瞄准京津冀、长三角、珠三角等发达地区，进一步完善优惠政策，重点引进装备制造、数据应用、新能源新材料等上下游关联产业。办好第三届蒙商大会、全国无人机系统大会等会展活动。强化要素保障。坚持“优势互补、错位发展”，统筹推进工业园区建设，补齐基础配套设施短板，推动产业向园区集中、要素向园区集聚。加快中国联通网络直联北京节点建设。进一步完善电力基础设施，开工察右后旗500千伏、巨宝庄500千伏变电站等项目。深入开展第三次全国国土调查，编制国土空间规划，合理界定城乡各类用地使用和权属状况。开展闲置用地清理，盘活工业用地资源，做好长期停产企业土地收储工作。严格调控新增建设用地指标，保障重大项目、民生工程用地需求。强化项目调度。实施白音察干至安业高速公路等重点项目，规划建设一批资源路、产业路、旅游路。加快乌兰察布机场升级国际货运机场，启动凉城、化德、四子王通用机场建设。实施中心城区抗旱应急备用水源工程，做好丰镇头道沟抗旱水源工程等项目前期工作。建立政府与民营企业沟通协调机制，实行领导干部包联重点企业和重大项目责任制，定期召开民营企业座谈会，每月召开经济调度会，一企一策、一项目一策，及时研究解决企业运行、项目落实中的困难和问题。围绕国家、自治区重点投向和全市产业规划，谋划储备一批重点项目，增强项目建设后劲。

　　（五）深入推进创城工作，提升高质量发展承载力

　　坚持规划引领，推进扩容提质。围绕城市定位和新兴产业发展需求，进一步健全城乡规划体系，编制高铁站区、霸王河以东片区城市设计，开展中心城区“产城融合”和“城市双修”专项规划编制工作，推进以人为核心的城市建设。加强市政基础建设。打通中心城区断头路。推进城镇污水、天然气和供水供热管网改造，建设建筑垃圾消纳厂和生活垃圾协同处置利用基地。推进“宽带乌兰察布”建设，嘎查村实现4G网络全覆盖。改善群众居住条件。把棚户区改造作为最大的民生工程，全力推动集宁区棚改项目，统筹推进各旗县棚户区改造，全市实施1.2万套棚改任务，让棚户区居民尽早住上新房、暖房、安心房。全面启动“半拉子”工程，新开工建设一批住宅项目。构建城市生态体系。加强城市园林绿化，实施重点区域公厕改造，全面完成南沙河河道综合治理、园林景观绿化、地下综合管廊等工程，建设人与自然和谐共生的美丽家园。

　　加强精细管理，提升创建水平。统筹推进老旧小区综合整治和节能改造，加大城乡结合部环境治理力度。开展“物业服务质量提升年”活动，出台物业服务企业信用等级评价办法，建立全市住宅专项维修资金统一管理平台，着力解决群众反映强烈的水电气暖和物业服务等问题。建设综合性数字化城市管理平台，提高城市智慧化管理水平。深入推进全国文明城市、国家食品安全示范城市、国家节水型城市、国家森林城市创建工作，国家级卫生旗县达到30%，自治区级卫生旗县实现全覆盖，全面提升城市宜居宜业水平。

　　（六）加强保障和改善民生，让群众共享高质量发展成果

　　坚持以人民为中心的发展思想，完善制度，守住底线，寓管理于服务之中，精心做好各项民生工作。

　　实施就业优先政策。落实援企稳岗补贴等政策措施，支持民营企业、小微企业更好发挥就业主渠道作用。实施多种模式的就业技能培训，统筹抓好高校毕业生、农民工、退役军人等重点群体就业。坚持以创业带动就业，完善百度、软通动力等孵化平台，扎实办好“双创”博览会，高质量建设“双创”示范基地、国家级众创空间和星创天地。强化治欠保支工作，切实维护好农民工合法权益。

　　办好人民满意的教育。深化教育领域综合改革，加强校长和教师队伍建设，全面推行中小学绩效工资制度。加强普惠性幼儿园建设，高度重视幼教师资力量培育，多渠道增加学前教育供给。推动义务教育城乡一体化发展，促进普通高中优质特色多样化发展。积极消除城镇学校“大班额”，保障好农民工随迁子女就学。深化产教融合、校企合作，完善现代职业教育体系。优先重点发展民族教育，统筹发展民办教育、特殊教育、高等教育，推进全民继续教育，建设学习型社会。加强与北京学校交流合作，促进优质教育资源共享。

　　推进文化科技创新体系建设。深入实施公民道德建设工程，大力弘扬社会主义核心价值观。深化国有艺术院团改革，加强乌兰牧骑建设，广泛开展文化惠民演出。实施重大文物保护工程，推进优秀传统文化传承保护。增加全社会科技投入，落实技术交易补助和税收优惠政策，激发科研人员的创新活力。培养和引进科技领军人才，引导高校和科研院所与我市优势产业深度对接，健全需求为导向、企业为主体的产学研一体化创新机制，支持中小企业创新，促进科技成果转化。

　　加快建设健康乌兰察布。深化医药卫生体制改革，全面推行医联体、医共体和远程医疗建设，推进分级诊疗、单病种付费和临床路径管理，扩大异地就医定点医疗覆盖面。二级以上医院全部实行绩效考核，力争40%的基层医疗卫生机构达到国家标准。大力发展蒙中医药事业。建立完善全民健康信息平台，普及应用居民电子健康卡。加快医养一体化项目建设，加强与北京三甲医院合作，探索医养结合新模式。推广居家养老、健康护理和旅居候鸟式养老等模式，推进养老服务市场化。加强公共卫生和重大疾病防治工作。丰富体育赛事活动，发展竞赛表演等体育产业。加快推进小型健身点建设，加大公共体育场馆设施向群众免费开放力度。推广中小学足球、网球、冰雪运动。

　　强化社会保障兜底功能。深入实施全民参保计划，提高困难群体参保率。推进机关事业单位养老保险制度改革，提高退休人员养老保险待遇。强化社保基金安全管理。完善职工和城乡居民医保制度，提升医疗保障水平。加强社会救助统筹，不断完善城乡低保、临时救助等制度，大力发展社会福利和慈善事业。全面排查城乡居民参加医保、享受低保情况，分类拿出解决措施，确保不缺不漏、应保尽保。

　　提升社会治理水平。扎实开展安全生产隐患排查治理和重点行业领域专项整治，下大力气抓好安全生产、食药安全和交通安全，坚决遏制重特大生产安全事故。完善市县应急管理平台建设，抓好气象服务、防汛抗旱、防灾减灾等工作。完善领导接访和包案工作机制，提高网上信访办理水平，加强矛盾纠纷排查化解，切实解决群众合理诉求。加强信息化、智能化、立体化社会治安防控体系建设，深入开展扫黑除恶专项斗争，建设平安乌兰察布“升级版”。加强全民普法教育，完善公共法律服务体系，健全社会信用体系。发挥社工和志愿者等社会力量作用。维护妇女、儿童、老年人、残疾人合法权益。

　　深入推进民族团结、边疆稳固。开展“全国民族团结进步示范市”创建工作，加快少数民族聚居区脱贫攻坚步伐，切实提高城市民族工作水平，推进蒙古语文规范化标准化信息化建设，促进各民族共同团结奋斗、共同繁荣发展。

　　支持军队和国防建设，加强退役军人服务管理工作，提高优军服务保障水平。

　　三、努力建设人民满意的政府

　　贯彻新思想、奋进新时代，对政府工作提出新的更高要求。我们要不忘初心、牢记使命，树牢“四个意识”，坚定“四个自信”，坚决做到“两个维护”，建设让党放心、让人民满意的政府。

　　加强法治政府建设。坚持“法定职责必须为、法无授权不可为”，牢固树立法治思维，切实把政府工作纳入法治轨道。依法接受人大及其常委会的法律监督和工作监督，自觉接受政协民主监督，主动接受社会舆论监督。全面推行权力清单制度，严格按程序决策、按规矩办事。充分听取各方面意见，落实政府法律顾问制度，推进科学民主决策。深入落实行政执法责任制，促进规范公正文明执法。加大政务公开力度，增强政府工作透明度，让权力在阳光下运行。建立新闻发布制度，及时回应社会关切。

　　加强效能政府建设。认真履职尽责，敢于担当、主动作为，以更严的要求、更高的标准提高政府执行力。按时完成政府机构改革，优化职能配置，提升行政效率。加强专项督查和跟踪问效，进一步完善正向激励和容错纠错机制，让改革者轻装上阵、担当者脱颖而出、实干者大显身手。整合市县两级非紧急热线电话，实行12345热线一号受理、一次性办结，打造统一便捷高效的企业和市民服务平台，提升政府公共服务水平。广泛听取各民主党派、工商联、无党派人士意见，积极支持工会、共青团、红十字会等群众团体开展工作。

　　加强廉洁政府建设。始终把廉洁从政作为政府工作的底线，坚定不移把反腐倡廉推向深入。主动接受纪检监察监督，强化审计监督。推进“两学一做”学习教育常态化制度化，开展“不忘初心、牢记使命”主题教育。严格执行中央八项规定及其实施细则和自治区配套办法，驰而不息整治“四风”。树立过“紧日子”思想，勤俭办一切事业，严控“三公”经费预算，优先保工资、保运转、保基础民生。建立政府“三保”预算审查机制。政府工作人员要廉洁修身，勤勉尽责，干干净净为人民做事。

　　各位代表！

　　只有干出来的精彩，没有等出来的小康，幸福总在奋斗的路上。我们坚信，只要凝聚同心筑梦的精神力量，激发砥砺奋进的责任担当，就一定能够在前进中成就梦想、铸就辉煌。让我们更加紧密地团结在以习近平同志为核心的党中央周围，大力弘扬一往无前的蒙古马精神，乘势而为、迎难而上，奋力谱写乌兰察布高质量发展新篇章，以优异成绩庆祝中华人民共和国成立70周年！
